# Modeling African American prostate adenocarcinoma by inducing defined genetic alterations in organoids

**DOI:** 10.18632/oncotarget.17230

**Published:** 2017-04-19

**Authors:** Kenji Unno, Meejeon Roh, Young A. Yoo, Yousef Al-Shraideh, Lu Wang, Larisa Nonn, Sarki A. Abdulkadir

**Affiliations:** ^1^ Department of Urology, Northwestern University Feinberg School of Medicine, Chicago, IL 60611, USA; ^2^ Department of Pathology, Northwestern University Feinberg School of Medicine, Chicago, IL 60611, USA; ^3^ Department of Pathology, University of Illinois at Chicago, Chicago, IL 60612, USA; ^4^ The Robert H. Lurie Comprehensive Cancer Center, Northwestern University Feinberg School of Medicine, Chicago, IL 60611, USA

**Keywords:** prostate cancer, organoid culture, malignant transformation, cancer development, African American

## Abstract

Genomic studies are rapidly identifying genetic alterations in human cancer, but functional validation of such alterations has been slow. Here, using human prostate cancer as a model, we have assessed the feasibility of engineering defined genetic alterations in well-known cancer driver genes to transform benign prostate epithelial organoids derived from African American men. Benign human prostate organoids were transduced with lentiviruses expressing MYC, shPTEN, shTP53 and AR, alone and in various combinations, to recapitulate prostate cancer development. Organoids expressing MYC, shPTEN, shTP53 and AR (denoted MPPA); MYC, shPTEN and shTP53 (MPP); or MYC (M) were significantly larger, had higher proliferation rates and demonstrated pathologically transformed morphology compared to organoids transduced with control lentivirus. Alterations in MYC, PTEN and TP53 also affected the rate of organoid basal-to-luminal differentiation *in vitro*. MPPA and MPP organoids expressed the clinical prostate cancer marker AMACR and developed prostate adenocarcinoma when grafted under the renal capsule in mice. These data indicate that genetic alterations commonly observed in human prostate cancer can be rapidly modeled in human organoid culture. Prostate cancer organoids provide a useful pre-clinical model for the evaluation of new candidate cancer genes, cancer disparities, and potentially for testing of novel therapeutic agents.

## INTRODUCTION

The burden of prostate cancer in many parts of the world including the United States is high, with approximately 180,890 new cases and 26,120 deaths expected in the United States in 2016 [1]. In particular, African American (AA) men have higher prostate cancer incidence and mortality than other populations [2]. Yet, there is a general paucity of *in vitro* prostate cancer models, including for African American tumors. Recent advances in *in vitro* human organoid methodology open a new pre-clinical model to study disease, including prostate cancer. Organoids are defined as a collection of organ-specific cell types derived from stem cells/progenitors that self-organize through spatially restricted lineage commitment in a manner that recapitulates *in vivo* organ development [3]. Human and mouse prostate organoids resemble normal prostate epithelial architecture with basal and luminal cells expressing of androgen receptor (*AR*) within 21 days [4, 5].

Organoids are grown in a three-dimensional controlled *in vitro* environment and have many advantages compared to other models. Organoids can be easily manipulated by using viruses, CRISPR-Cas9, and inhibitors [4–7]. Functional studies in 2D cell line models are fraught with many shortcomings that render them of limited utility, while *in vivo* models using genetically engineered animals are expensive and time consuming. As prostate organoids are derived from normal human prostate epithelial cells, these cells are idea to engineer specific genetic alterations in candidate cancer genes, and thus could provide a rapid assay for functional assessment in a relevant *in vitro* cancer models [3–5].

Individual heterogeneity, genetic complexity and adaptations in signaling pathways in prostate tumors constitute major hurdles in investigating molecular mechanisms and targeted therapy. The MYC/PTEN/TP53 pathway has been implicated as a major pathway for development of lethal human prostate cancer. *MYC* overexpression and *PTEN* loss in the younger men, and additional loss of *TP53* in the older men were correlated with a poor survival outcome [8], while *AR* was significantly amplified in castration-resistant prostate cancer (CRPC) [9]. *TP53* is mutated at the highest frequency in prostate cancer cells followed by *PTEN* gene mutation (http://cancer.sanger.ac.uk) [10]. Human prostate cancers with transcriptional gene signatures indicative of MYC activation, *PTEN* loss and *TP53* loss are associated with a 3.2-fold higher risk of death [8]. Notably, this effect was present even in patients with low- to-intermediate Gleason scores of 6 and 7, and reproducible in an independent patient cohort. Cancers with the MYC+/PTEN-/TP53- signature were more aggressive, with a shorter time to disease recurrence after primary treatment [8]. These findings mirrored results from conditional *MYC+*;*Pten*-mutant;*Tp53*-mutant transgenic mice, where stepwise alterations in *MYC, Pten* and *Tp53* led to the development of advanced cancer [11, 12].

In the current study, we sought to characterize prostate organoids generated from African American subjects that were engineered to express combination of *MYC, AR, shPTEN* and *shTP53*. These genetically engineered organoids became transformed *in vitro* and formed prostate cancer *in vivo*, validating organoid cultures as a model to study prostate tumorigenesis.

## RESULTS

### Establishment of African American prostate organoids with altered expression of *MYC*, *PTEN*, *TP53* and *AR*

**Figure 1:**
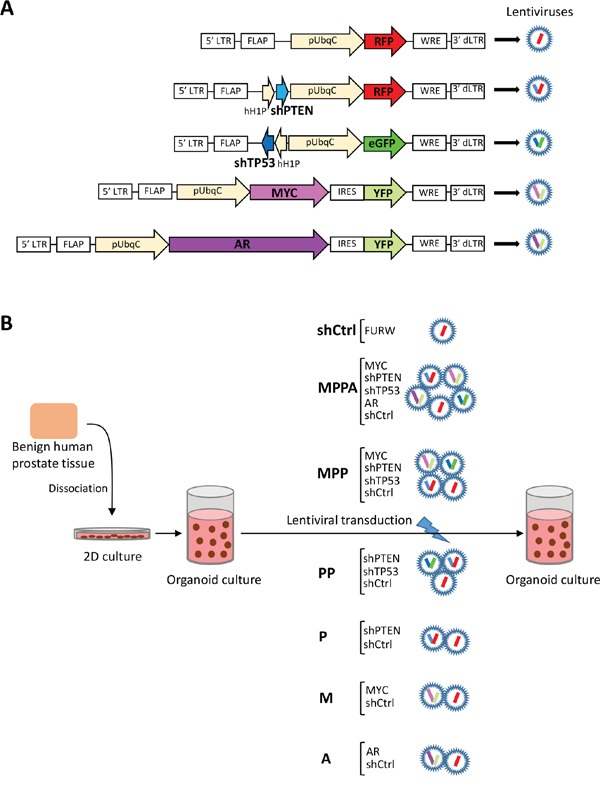
Schematic representation of experimental overview **(A)** Lentiviral vectors expressing RFP alone, shPTEN and RFP, shTP53 and eGFP, MYC and YFP, and AR and YFP. dLTR, deleted long-terminal repeat; FLAP, nucleotide segment that improves transduction efficiency; WRE, woodchuck hepatitis virus post-transcriptional regulatory element; pUbqC, ubiquitin promoter; hH1P, H1 promoter; IRES, internal ribosome entry site. **(B)** Schematic representation of organoid culture of human primary epithelial cells with lentiviral transduction. MPPA (MYC, shPTEN, shTP53, and AR-transduced organoids); MPP (MYC, shPTEN, and shTP53-transduced organoids); PP (shPTEN and shTP53-transduced organoids); P (shPTEN-transduced organoids); M (MYC-transduced organoids); A (AR-transduced organoids). FURW is control vector which expresses only RFP.

We first established an organoid culture system using benign human prostate epithelial cells *in vitro*. These cells formed organoid structures by day 8 consisting of basal (CK5+), intermediate (CK5+/CK8+) and luminal (CK8+) cells (Supplementary Figure 1). By day 21, expression of cytokeratin 8+ luminal cells was increased indicating differentiation. Organoids also expressed AR and PSA. Next, we sought to develop prostate cancer organoids *in vitro*. The *MYC* oncogene and the tumor-suppressor genes *PTEN* and *TP53* are often altered in human prostate cancer, while *AR* amplification and overexpression has been implicated in CRPC [8, 9]. We modeled alterations in *MYC*, *PTEN*, *TP53*, and *AR* either alone or in combination by lentiviral-mediated delivery of oncogene cDNAs for overexpression or tumor-suppressor shRNA for knockdown. The lentiviral vectors co-expressed red fluorescent protein, RFP (*shPTEN, control*), enhanced green fluorescent protein, eGFP (*shTP53*), yellow fluorescent protein, YFP (*MYC* and *AR*) (Figure 1A). Fluorescence signal was used to confirm transduction efficiency. To examine the complementary effect of the genetic alterations, we generated the following organoids (Figure 1B): *MYC/shPTEN/shTP53/AR* (MPPA); *MYC/shPTEN/shTP53* (MPP); *shPTEN/shTP53* (PP); *shPTEN* (P); *MYC* (M); *AR* (A); and empty vector (*shCtrl*). In our method, we first expanded benign human prostate epithelial cells from four African American subjects (AA-1, AA-2, AA-3, and AA-4) and a non-African American subject in two-dimensional cell culture (Figure 1B). Prior to organoid culture, CK5 single positive basal cells and CK5/CK8 double positive intermediate cells were determined in all subjects (Supplementary Figure 2).

**Figure 2:**
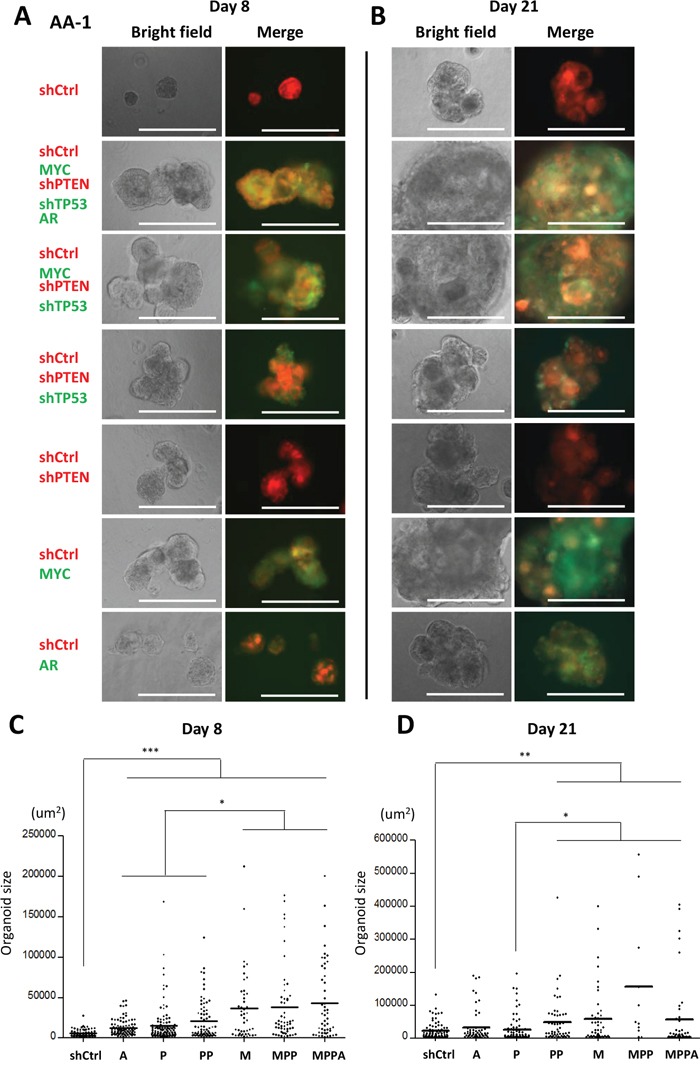
Engineered organoids derived from African American subject AA-1 (**A** and **B**) Representative bright field and fluorescence images of MPPA, MPP, PP, P, M, A, and shCtrl organoids at day 8 (A) and day 21 (B). Scale bars 300 um. (**C** and **D**) Organoid sizes (areas) at day 8 (C) and day 21 (D). The areas (um^2^) of organoids >25 um diameter were measured by ImageJ. *p < 0.05, **p < 0.01, ***p < 0.001.

**Figure 3:**
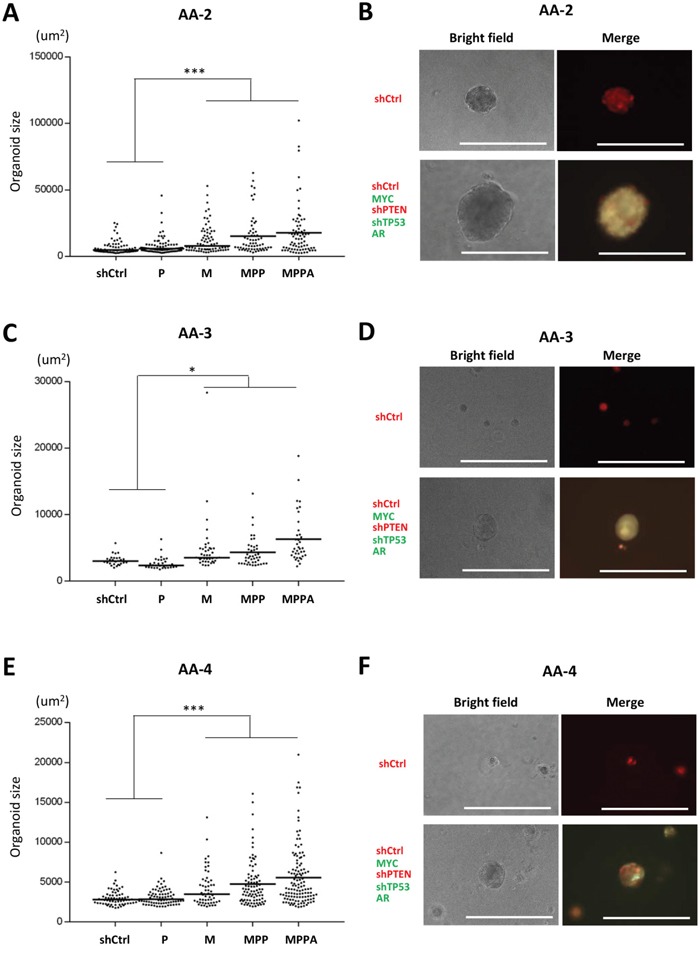
Engineered organoids derived from additional African American subjects AA-2, AA-3, and AA-4 (**A, C**, and **E**) Organoid sizes (areas) of AA-2 (A), AA-3 (C), and AA-4 (E) at day 8. The areas (um^2^) of organoids >25 um diameter were measured by ImageJ. *p < 0.05, ***p < 0.001. (**B, D**, and **F**) Representative bright field and fluorescence images of MPPA (MYC, shPTEN, shTP53, and AR-transduced) and shCtrl organoids of AA-2 (B), AA-3 (D), and AA-4 (F) at day 8. Scale bars 300 um.

We initially generated organoids from an African American (AA-1) and a non-African American subject (Figure 2 and Supplementary Figure 3). In these experiments, MPPA, MPP and M organoids were all significantly larger than control organoids at day 8 and day 21. We next generated organoids from 3 additional AA subjects (AA-2, AA-3, and AA-4). Consistently, MPPA, MPP, and M organoids were significantly larger than shCtrl organoids (Figure 3), although some variation in transformed organoid sizes was apparent, with AA-1 and AA-2 organoids being larger overall than AA-3 and AA-4 organoids (Figure 2 and 3). This may reflect differences in intrinsic susceptibility to transformation between cells derived from different individuals. The impact of genetic background will need a more comprehensive exploration in the future studies.

**Figure 4:**
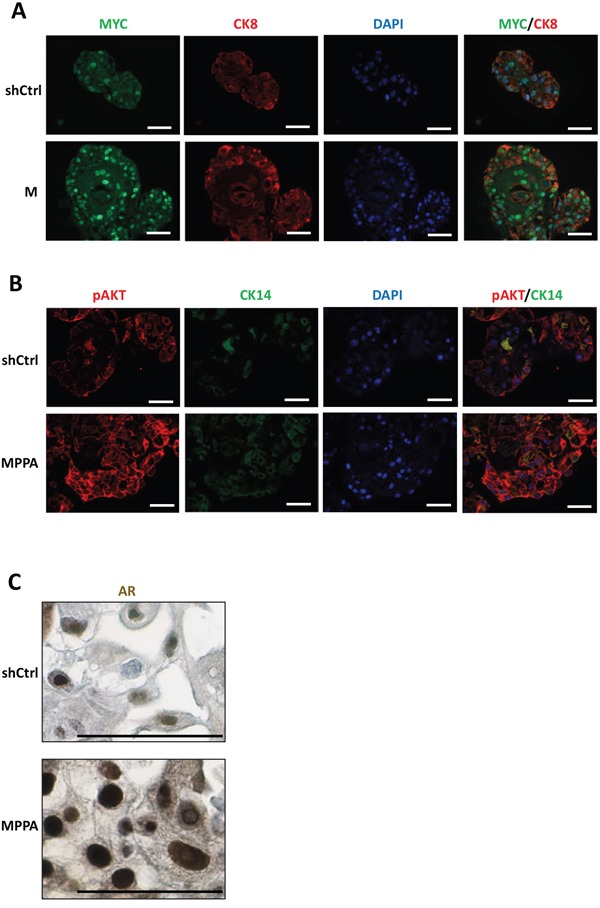
Assessment of MYC, p-AKT, and AR expressions in organoids **(A to C)** Representative images of MYC, AR, and p-AKT expressions. Organoids with immunofluorescence staining for MYC (green) and CK8 (red) at day 8 (A), pAKT (red) and CK14 (green) at day 21 **(B)**, and IHC staining for AR at day 21 (C). Blue, DAPI. Scale bars 50 um.

**Figure 5:**
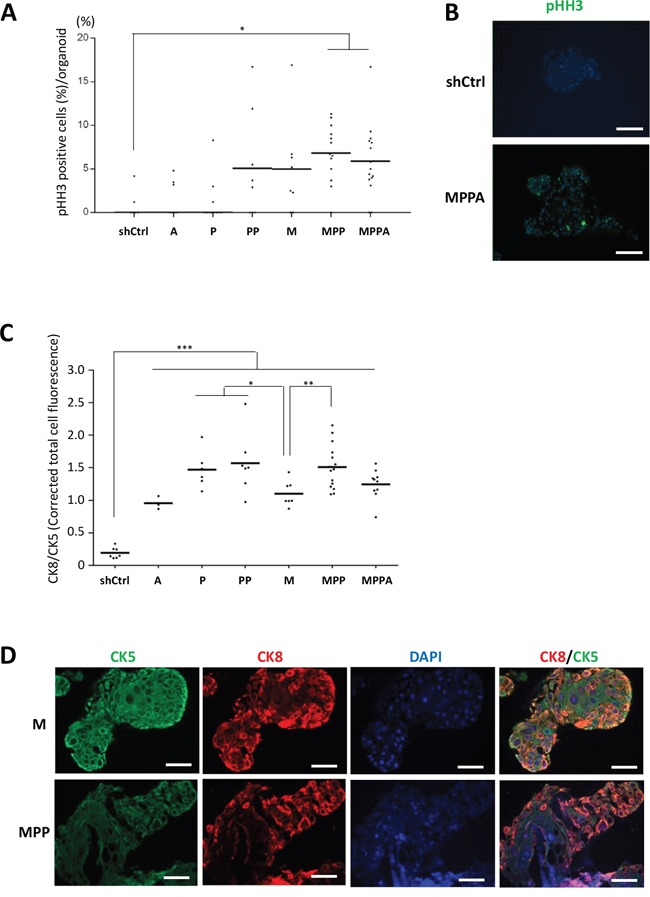
Analyses of proliferation and differentiation in organoids **(A)** pHH3 positive cells (%) per organoid at day 8. **(B)** Representative images of MPPA and shCtrl organoids with immunofluorescence staining for pHH3 at day 8. Scale bars 100 um. **(C)** Corrected total cell fluorescence (CTCF) of CK8 and CK5 was measured by ImageJ. CTCF of CK8 was divided by CTCF of CK5 at day 8, indicating differentiation criterion. **(D)** Representative images showing difference of M and MPP organoid differentiations with immunofluorescence staining for CK8 (red) and CK5 (green) at days 8. Scale bars 50 um. Blue, DAPI. *p < 0.05, **p < 0.01, ***p < 0.001.

Examination of organoids by immunohistochemistry confirmed overexpression of nuclear MYC and AR and membrane p-AKT (as a marker of PTEN depletion) in the appropriate organoids (Figure 4). Phospho-histone H3 (pHH3) staining was used to assess the mitotic index in the organoids. The pHH3 mitotic indices were significantly higher in MPPA and MPP organoids, with M and PP organoids showing a trend in increase in mitotic index (Figure 5A and 5B). The mitotic indices correlated well with organoid size.

### Oncogenic alterations accelerate luminal cell differentiation of organoids

The influence of genetic alterations on epithelial differentiation of organoids was examined by the expression of the basal cell marker CK5 and the luminal cell marker CK8. At day 8, MPPA, MPP, PP, P, M, and A organoids had more luminal cell differentiation than shCtrl organoids as shown by a higher ratio of CK8 to CK5 expression (Figure 5C). CK5 expression of M organoids was significantly higher than that of MPP, PP, and P organoids (Figure 5C and 5D). Thus the genetic oncogenic alterations examined here all promoted luminal differentiation in organoid culture.

### Oncogenic alterations transform organoids *in vitro*

**Figure 6:**
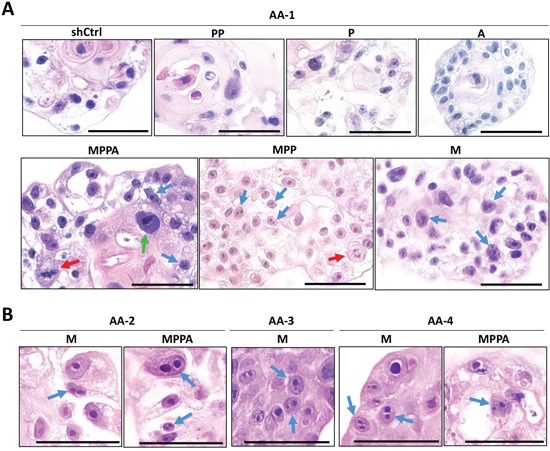
Assessment of malignant transformation of organoids *in vitro* Representative images of MPPA, MPP, PP, P, M, A, and shCtrl organoids derived from AA-1 sample with H&E staining at day 8 (red arrows, mitotic cells; blue arrows, multiple prominent nucleoli; green arrow, enlarged nuclei and prominent nucleoli) **(A)**. Representative images of multiple prominent nucleoli of MPPA and M organoids derived from AA-2, AA-3, and AA-4 tissues with H&E staining at day 8 (blue arrows, multiple prominent nucleoli) **(B)**. Scale bars 50 um.

**Figure 7:**
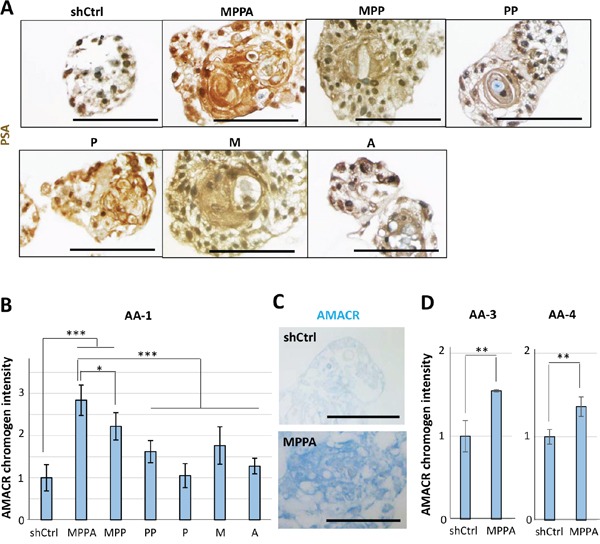
Immunohistochemical staining for prostate-specific antigen (PSA) and α-methylacyl-CoA racemase (AMACR) in organoids **(A)** Representative images of MPPA, MPP, PP, P, M, A, and shCtrl organoids derived from AA-1 tissue with IHC staining for PSA at day 8. **(B)** Relative AMACR chromogen intensity of MPPA, MPP, PP, P, M, A, and shCtrl organoids derived from AA-1 at day 21. Chromogen intensity was measured by Fiji software (ImageJ) (http://fiji.sc/Fiji). **(C)** Representative images of MPPA and shCtrl organoids with IHC staining for AMACR at day 21. **(D)** Relative AMACR chromogen intensity of MPPA and shCtrl organoids derived from AA-3 and AA-4 at day 8. Scale bars 100 um. *p < 0.05, **p < 0.01, ***p < 0.001.

Histological analysis of AA-1 organoids by H&E staining showed that MPPA, MPP, and M organoids exhibited cells with enlarged nuclei, prominent nucleoli, and increased mitosis (Figure 6A), features that are consistent with transformation. Interestingly, multiple prominent nucleoli were remarkably observed in cells of MPPA, MPP, and M organoids (Figure 6A). Likewise, multiple prominent nucleoli were noticeable in MPPA and M organoids of other AA subjects (Figure 6B). PSA was positive in all organoids, with varying levels of expression, confirming AR activation (Figure 7A). To further assess transformation, the expression of α-methylacyl-CoA racemase (AMACR), a clinical marker of prostate adenocarcinoma, was examined. AMACR expression was significantly increased in MPPA and MPP organoids relative to controls (Figure 7B and 7C). Furthermore, AMACR expression of MPPA organoids was significantly higher than other organoids (Figure 7B). These results support transformation of organoids *in vitro* by MYC, shPTEN and shTP53. Similarly, in organoids derived from other AA subjects, AMACR expression was significantly increased in MPPA organoids relative to controls (Figure 7D).

### The response to allosteric AKT inhibitor, MK-2206, of organoids

**Figure 8:**
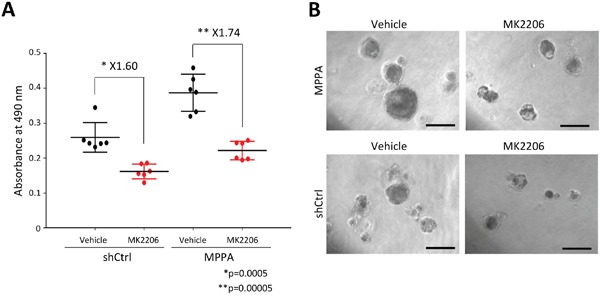
Drug treatment response in organoids **(A)** The response to allosteric AKT inhibitor, MK-2206, of MPPA and shCtrl derived from AA-1 tissue was assessed by MTS-based cell proliferation assay. **(B)** Representative images of MPPA and shCtrl organoids at days 6 after 3 uM MK-2206 and DMSO (vehicle) treatment. Scale bars 100 um.

Since MPPA organoids shows high p-AKT signal by knockdown of PTEN, we next assessed the sensitivity of MPPA organoids to AKT inhibitor, MK-2206. After organoids formed, we treated MK-2206 to MPPA and shCtrl organoids for 6 days. The viability of MPPA organoids significantly decreased relative to vehicle treatment (Figure 8A). However, shCtrl organoids were also affected by MK-2206 (Figure 8A and 8B). These results suggest that MPPA organoids may allow us to investigate novel targeted therapies for altered expression of *MYC*, *PTEN*, *TP53* and *AR*.

### MPPA and MPP organoids form prostate cancer *in vivo*

**Figure 9:**
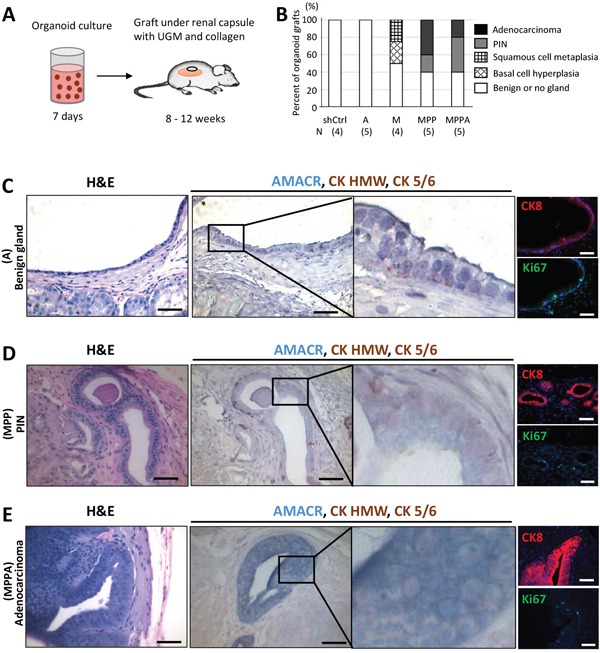
*In vivo* tumorigenicity of transformed organoids **(A)** Schematic representation of the process of MPPA, MPP, M, A, and shCtrl organoid transplants under the renal capsule of NOD/SCID mice. **(B)** Pathological analyses of organoid grafts. Grafts were stained with H&E, AMACR, CK HMW, and CK 5/6, and analyzed by a pathologist. N, number of samples analyzed. **(C)** Benign gland developed from A organoid transplants. Images with H&E staining; IHC staining for AMACR, CK HMW, and CK 5/6; and immunofluorescence staining for CK8 and Ki67. Blue, DAPI. **(D)** PIN developed from MPP organoid transplants. Images with H&E staining; IHC staining for AMACR, CK HMW, and CK 5/6; and immunofluorescence staining for CK8 and Ki67. Blue, DAPI. **(E)** Adenocarcinoma developed from MPPA organoid transplants. Images with H&E staining; IHC staining for AMACR, CK HMW, and CK 5/6; and immunofluorescence staining for CK8 and Ki67. Blue, DAPI. Scale bars 50 um.

To assess the phentoype of *in vitro* transformed organoids *in vivo*, we transplanted AA-1 derived shCtrl, MPPA, MPP, M, and A organoids with rat urogenital sinus mesenchyme (UGM) under the renal capsule of immunodeficient mice. We transplanted organoids cultured for 7 days with UGM under the renal capsule (Figure 9A). Histological analysis showed that all shCtrl and A grafts were normal, while 60% of MPPA and MPP organoid grafts formed PIN or adenocarcinoma (Figure 9B, 9C, 9D, and 9E). PIN lesions expressed AMACR and basal cytokeratins, while adenocarcinoma glands expressed AMACR without basal cytokeratins (Figure 9D and 9E). 50% of M organoids revealed basal cell hyperplasia and squamous cell metaplasia (Supplementary Figure 4A and 4B). Basal cell hyperplasia lesions were uniformly positive for the basal cell cytokeratins with the outer layers negative for CK8 while inner cells were CK8 positive, indicating that outer and inner cells were basal and intermediate cells, respectively. In addition, the outer cell layer was actively proliferating (Supplementary Figure 4A). Squamous cell metaplasia lesion cells from the M organoid graft expressed basal cell cytokeratins with an inner layer that is also CK8 positive (Supplementary Figure 4B).

## DISCUSSION

The purpose of this study was to transform normal prostate cells with defined genetic alterations into prostate cancer *in vitro* with a focus on African American samples. Overexpression of MYC, with knockdown of PTEN, TP53 with or without AR expression (MPPA or MPP) are able to transform organoids *in vitro* and these transformed organoids can form prostate cancer *in vivo* as determined by histology and expression of the clinical marker AMACR with lack of basal cells. It is notable that there is a good correlation between features of transformation *in vitro* (e.g. organoid size, proliferation, nuclear morphology, AMACR expression) and *in vivo* tumorigenicity in the tissue recombination assay.

For the first time to our knowledge, we established benign and transformed prostate organoids from African American subjects. Interestingly, there was heterogeneity in the size of organoids from different individuals, perhaps reflecting intrinsic (germline) variations affecting organoid growth and tumor susceptibility. Considering the higher prostate cancer incidence and mortality in African American men, further studies patterned on ours could enhance our understanding of racial and ethnic disparities in prostate cancer.

In this study, we attempted to transform four African American derived benign prostate epithelial cells and succeeded to develop malignant AA organoids for all of them. Among AA malignant organoids denoted as MPPA, AA-1 and AA-2 MPPA organoids were larger than AA-3 and AA-4 MPPA organoids. Regardless of organoid size differences, MPPA organoids derived from all AA subjects showed multiple prominent nucleoli. To understand variety and identity of AA tissue derived malignant organoids, genetic and epigenetic analyses of AA benign prostate tissues will be needed in future studies.

Our findings are similar to a recent report showing that coexpression of MYC and constitutively active myrAKT1 (which mimics the effect of *PTEN* knockdown) transforms human prostate basal and luminal cell derived organoids [13]. The MYC/PTEN/P53 pathway has been shown in mouse models and human patients to be associated with advanced lethal prostate cancer [8, 11]. Therefore the identification of therapeutic strategies against this pathway will be of high significance. The development of organoids reflecting these genetic changes could facilitate screening efforts to identify agents that can inhibit these tumors.

Notably, MYC/myrAKT1-transduced basal or luminal cell derived organoids developed tumors composed of a mixture of adenocarcinoma with squamous cell carcinoma or adenosquamous carcinoma *in vivo*, respectively. Some MYC and myrAKT1-transduced luminal organoids showed adenosquamous differentiation in organoid culture [13]. Our own MYC organoids also showed squamous differentiation and basal cell hyperplasia. Squamous cell carcinoma and adenosquamous carcinoma of the prostate are very rare in human patients, with incidence of <1% of all prostate carcinomas [14]. Since we did not observe squamous cell differentiation in our organoids with TP53 knockdown, this may suggest that *TP53* loss inhibits squamous differentiation. Other studies have shown that depletion of p53 leads to induction of the androgen receptor [15, 16]. Although organoid-culture has been recognized as advanced 3D culture system, it is still far from *in vivo* environment in terms of nutrition, the existence of cancer-associated fibroblasts, and surrounding structure. Further optimization of organoid-culture system may make it possible to develop more ideal organoids similar to the *in vivo* environment.

In summary, we have successfully established human prostate organoids in culture from African American subjects and have modeled *MYC*, *PTEN*, *TP53*, and *AR* alterations either alone or in combination to develop prostate cancer. This approach can facilitate the generation and analysis of a larger panel of normal and transformed organoids from diverse racial and ethnic backgrounds for studying prostate cancer and the investigation of novel targeted therapies.

## MATERIALS AND METHODS

### Ethics statement

Investigation has been conducted in accordance with the ethical standards and according to the Declaration of Helsinki and according to national and international guidelines and has been approved by the authors' institutional review board.

### Tissue collection and cell preparation

Primary human epithelial cells were isolated from radical prostatectomy tissues from prostate cancer patients at the University of Illinois at Chicago Medical Center according to guidelines and approval by the Institutional Review Board with written informed consent obtained from all patients. Fresh tissue from the peripheral zone was selected and excised with a 5 mm punch by a pathologist. Final pathology of the tissue was determined by H&E on a thin slice of the punch. The area must be 100% benign to have that classification. Isolation of prostate epithelial cells was as previously described [17, 18] based on the method developed by Donna Peehl [19]. Briefly, tissues were digested with collagenase and plated on collagen-coated dishes in PrEGM (Lonza, Walkersville, MD) for epithelial cell (PrE) growth. Cell type was validated by qRT-PCR for the expression of known basal epithelial cell markers (CK5+, p63+, AR-).

### Lentiviral vectors and preparation

FURW and FURW-shPTEN were kindly provided by Dr. Bryan W Luikart. FUGW-shTP53 was constructed as follows. After annealing of shTP53 sense; 5’-CTAGAGACTCCAGTGGTAATCTACTTCAAGAGAGTAGAT TACCACTGGAGTCTTTTTG-3’ (underline; part of XbaI, loop, part of BamHI) and antisense; 5’-GATCCAAAAAGACTCCAGTGGTAATCTACTCTCTTGAAGTAGATTACCACTGGAGTCT-3’ (underline; part of BamHI, loop, part of XbaI), double strand shTP53 sequence was ligated into pFUGW-H1 empty vector (Addgene #25870) at XbaI and BamHI sites. shTP53 sequence was referred to shRNA sequence of shp53 pLKO.1 puro plasmid (Addgene #19119). For FM1-MYC1 (full length MYC)-YFP construct, using primers; 5’-GGCCGGATCCGAGACGCTGGATTTTTTTCGGG-3’ (underline; BamHI) and 5’-TGGGGCCGCTAGCTTTACGCACAAGAGTTCCGTAG-3’ (underline; NheI), human full length MYC was amplified by PCR from FUW-tetO-hMYC template plasmid (Addgene #20723). PCR fragment was ligated into FM1 plasmid (kindly provided by Dr. Jeffrey Milbrandt) at BamHI and NheI sites. For FM1-AR-YFP construct, using primers; 5’-ATTAAAGGATCCACCATGGAAGTGCAGTTAGGGCTG-3’ (underline; BamHI) and 5’-TATGCCGCTAGCTTCACTGGGTGTGGAAATAGAT-3’ (underline; NheI), AR was amplified by PCR from pcDNA3.1-AR template plasmid (kindly provided by Dr. Jindan Yu). PCR fragment was ligated into FM1 plasmid at BamHI and NheI sites. All inserted fragments were validated by sequencing. Lentiviruses were prepared as previously described [20]. In order to calculate lentiviral titers, we infected HEK293T cell line with lentivirus expressing RFP (FURW and FURW-shPTEN), eGFP (FUGW-shTP53), or YFP (FM1-MYC1-YFP and FM1-AR-YFP). Titer is expressed as transducing units (TU)/ml calculated from RFP-, eGFP-, or YFP- positive cells (%) measured by flow cytometer.

### Organoid culture and lentiviral transductions

50,000 cells of benign human prostate epithelial cells were cultured in PrEGM media (LONZA, #CC-3165 & CC-4177) containing primocin (Invivogen, #ant-pm-1) in 10 cm dish. When cells were 50-70% confluent (approximately 7-9 days), cells were passaged once to expand cells and seeded at 10% confluent in PrEGM media containing primocin in 10 cm dishes. When cells were 50% - 70% confluent (approximately 4 days), we started organoid culture as described in detail previously [4]. After prostate epithelial cells were cultured for 4 days, cells were trypsinized to single cells. FURW, FURW-shPTEN, FM1-MYC1-YFP, FUGW-shTP53, and FM1-AR-YFP lentiviral vectors were used for shCtrl (control), PTEN knockdown, MYC overexpression, TP53 knockdown, and AR overexpression, respectively. Lentiviral infection was performed as follows. For MPPA organoids, shCtrl-, shPTEN-, MYC-, shTP53-, and AR-lentiviral infection was performed with each 10 multiplicity of infections (MOIs). For MPP organoids, shCtrl-, shPTEN-, MYC-, and shTP53-lentiviral infection was performed with 20, 10, 10, and 10 MOIs, respectively. For PP organoids, shCtrl-, shPTEN-, and shTP53-lentiviral infection was performed with 30, 10, and 10 MOIs, respectively. For P organoids, shCtrl-, and shPTEN-lentiviral infection was performed with 40, and 10 MOIs, respectively. For M organoids, shCtrl-, and MYC-lentiviral infection was performed with 40, and 10 MOIs, respectively. For A organoids, shCtrl-, and AR-lentiviral infection was performed with 40, and 10 MOIs, respectively. For shCtrl organoids, shCtrl-lentiviral infection was performed with 50 MOI. Lentiviral infection in prostate epithelial cells was performed with 8 ug/ml polybrene by centrifugation at 1,200 rpm for 3 hrs at room temperature (RT). Lentivirus-mediated transduced cells were seeded to organoid culture media. At days 8 and 21 after lentivirus-mediated transduction, organoids were analyzed. Organoids were passaged up to five times.

### Tissue recombination and renal capsule grafting

The procedure for tissue recombination and renal grafting was followed as described previously [4] with modification and was approved by the Institutional Animal Use and Care Committee (IACUC) of Northwestern University. Briefly, after lentivirus-mediated transductions, organoids were cultured for 7 days in organoid culture media. Organoids from one well of a 96-well low attachment plate were mixed with 250,000 dissociated rat urogenital sinus mesenchyme (UGM) cells from embryonic day 18 rat embryos. Mixture of organoids and UGM was resuspended in 20 ul of 3:1 collagen/setting buffer solution. The recombinants were cultured overnight in DMEM with 10% FBS and 10 nM DHT, followed by grafting under the renal capsules of male NOD/SCID mice (6-8 weeks). DHT pellet was implanted subcutaneously. Grafts were harvested at 8 and 12 weeks for analysis.

Mice are euthanized when they are not grooming, are lethargic, or otherwise exhibiting moribund behavior, or have Body Condition Scoring (BCS) of 2 or less.

### Drug treatment of organoids

AA-1 derived MPPA and shCtrl organoids were dissociated at passages 3 in organoid culture. Resuspended cells in organoid culture media were seeded to 96-well low attachment plate at 5,000 cells per 100 ul media. MPPA- and shCtrl- dissociated cells were seeded to each 12 wells. After organoids formed at days 4, 100 ul organoid culture media with 6 uM MK-2206 (final 3 uM) or DMSO (vehicle) was added to 100 ul culture (each 6 wells). At 3 days after drug treatment, additional 100 ul organoid culture media with 3 uM MK-2206 or DMSO was added to culture. At days 6 after drug treatment, representative bright field images were taken and viability was measured by CellTiter 96® AQ_ueous_ One Solution Cell Proliferation Assay (MTS) (Promega, Cat#G3581).

### Histology and immunofluorescence staining

Organoids were fixed with 10% formalin solution for 1 hr at RT. After centrifugation, organoids were solidified with histogel (Thermo Scientific Cat#HG-4000-012) on the biopsy-pads in the tissue-cassettes, incubated for 10 min in 30% ethanol, for 10 min in 50% ethanol, and overnight in 70% ethanol. Grafts were fixed with 10% formalin solution overnight at 4 °C, followed by incubation overnight at 4 °C in 70% ethanol. Paraffin-embedded sections (4 um) were deparaffinized and stained using hematoxylin and eosin (H&E) or specific antibodies. The following primary antibodies were used; mouse monoclonal anti-cytokeratin 8 1:500 (Covance, Cat#MMS-162P), rabbit polyclonal anti-cytokeratin 5 1:500 (Covance, Cat#PRB-160P), rabbit polyclonal anti-c-Myc 1:50 (Santa cruz, Cat#sc-764), rabbit monoclonal anti-pAKT 1:50 (Cell Signaling, Cat#4060), mouse monoclonal anti-CK14 1:400 (BioGenex, Cat#MU146-UC), rabbit polyclonal anti-pHH3 1:500 (Millipore, Cat#06-570), rat monoclonal anti-Ki67 1:100 (eBioscience, Cat#14-5698-80), rabbit polyclonal anti-PSA 1:300 (DAKO, Cat#A0562), and rabbit polyclonal anti-AR 1:200 (Thermo Scientific, Cat#RB-9030-P1). Sections were incubated in citrate buffer (pH 6) for antigen retrieval, followed by blocking with 10% normal goat serum (NGS) (Vector Labs, Cat#S-1000) and 5% bovine serum albumin (BSA), incubated with primary antibodies diluted in 5% NGS and 1% BSA. Sections were incubated with secondary antibodies. For immunofluorescence, following secondary antibodies were used; anti-mouse Alexa 594 1:400 (Invitrogen, Cat#A11005), anti-rabbit Alexa 488 1:400 (Invitrogen, Cat#A11008), anti-rabbit Alexa 594 1:400 (Invitrogen, Cat#A11012), anti-mouse Alexa 488 1:400 (Invitrogen, Cat#A11001), and anti-rat Alexa 488 1:400 (Invitrogen, Cat#A11006). Sections were counterstained with DAPI and mounted with ProLong Diamond Antifade Mountant (Molecular Probes, Cat#P36961). For 3,3’-diaminobenzidine (DAB) immunohistochemistry, after incubation in citrate buffer, sections were incubated in 3% H_2_O_2_ to block peroxidase. Biotinylated anti-rabbit 1:200 (Vector Labs, BA-1000) was used as a secondary antibody, followed by incubation with ABC reagent (Vector Labs, PK-7100), and incubation with 3,3’-diaminobenzidine (Sigma, Cat#D4168-50SET). Sections were counterstained with hematoxylin, dehydrated, and mounted with Cytoseal 60 (Richard-Allan Scientific, Cat#8310-4). For AMACR, CK HMW, and CK 5/6 staining; DuoFLEX Cocktail Anti-AMACR, Anti-Cytokeratin HMW, Anti-Cytokeratin 5/6 Ready to Use (DAKO, Cat#IC004) and Lab Vision MultiVision Polymer Detection System containing anti-mouse-HRP and anti-rabbit-AP (Thermo Scientific Cat#TL-012-MHRA) were used. The procedure for staining was performed followed by manufacturer's instructions, except primary and secondary antibodies incubation time to 1 hr. For AMACR single staining of organoids, manufacturer's instruction of Lab Vision MultiVision Polymer Detection System was modified as follows. Sections were incubated for 20 min with LVBlue solutions in AP-buffer. LVRed solutions were not added for eliminating CK HMW and CK 5/6 detection. Primary and secondary antibodies were incubated for 1 hr. Images were captured on a Leica DM5000B Microscope.

## SUPPLEMENTARY MATERIALS FIGURES


