# Supplementary material for: Modeling African American prostate adenocarcinoma by inducing defined genetic alterations in organoids
Source: Oncotarget. 2017 Apr 19;8(31):51264–76. doi: 10.18632/oncotarget.17230 (PMC5584247; doi:10.18632/oncotarget.17230)
Supplement: Supplementary file 1 [file oncotarget-08-51264-s001.pdf]

Modeling African American prostate adenocarcinoma by inducing defined genetic alterations in organoids

SUPPLEMENTARY MATERIAL

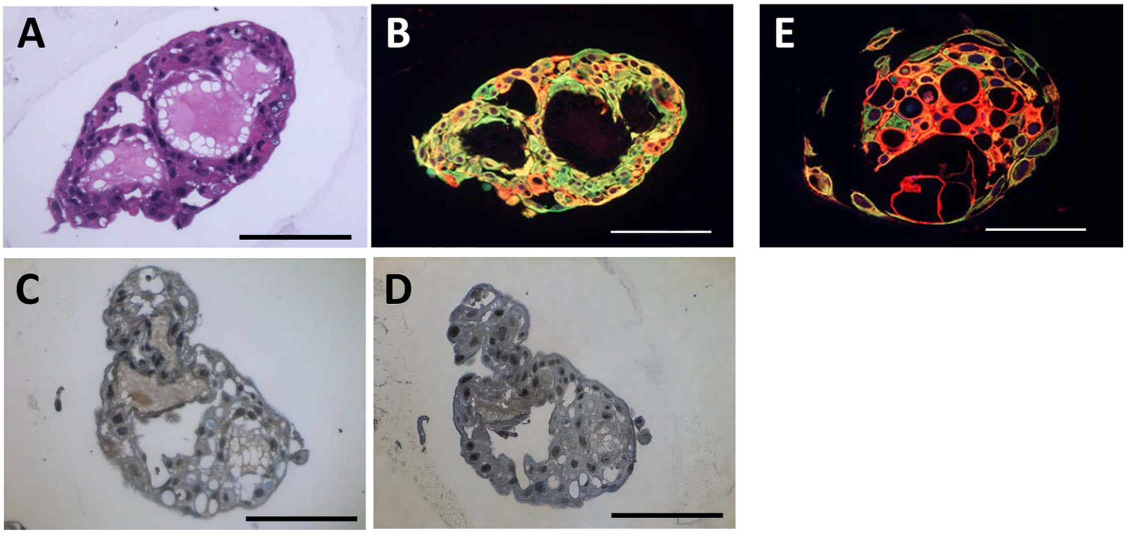

Supplementary Figure 1: Establishment of human prostate organoids from benign prostate epithelial cells. **(A to E)** Representative images of human prostate organoids with H&E **(A)**, immunofluorescence staining for CK5 (green) and CK8 (red) **(B)**, IHC staining for PSA **(C)** and AR **(D)** at day 8, and with immunofluorescence staining for CK5 (green) and CK8 (red) **(E)** at day 21. Scale bars 100 um.

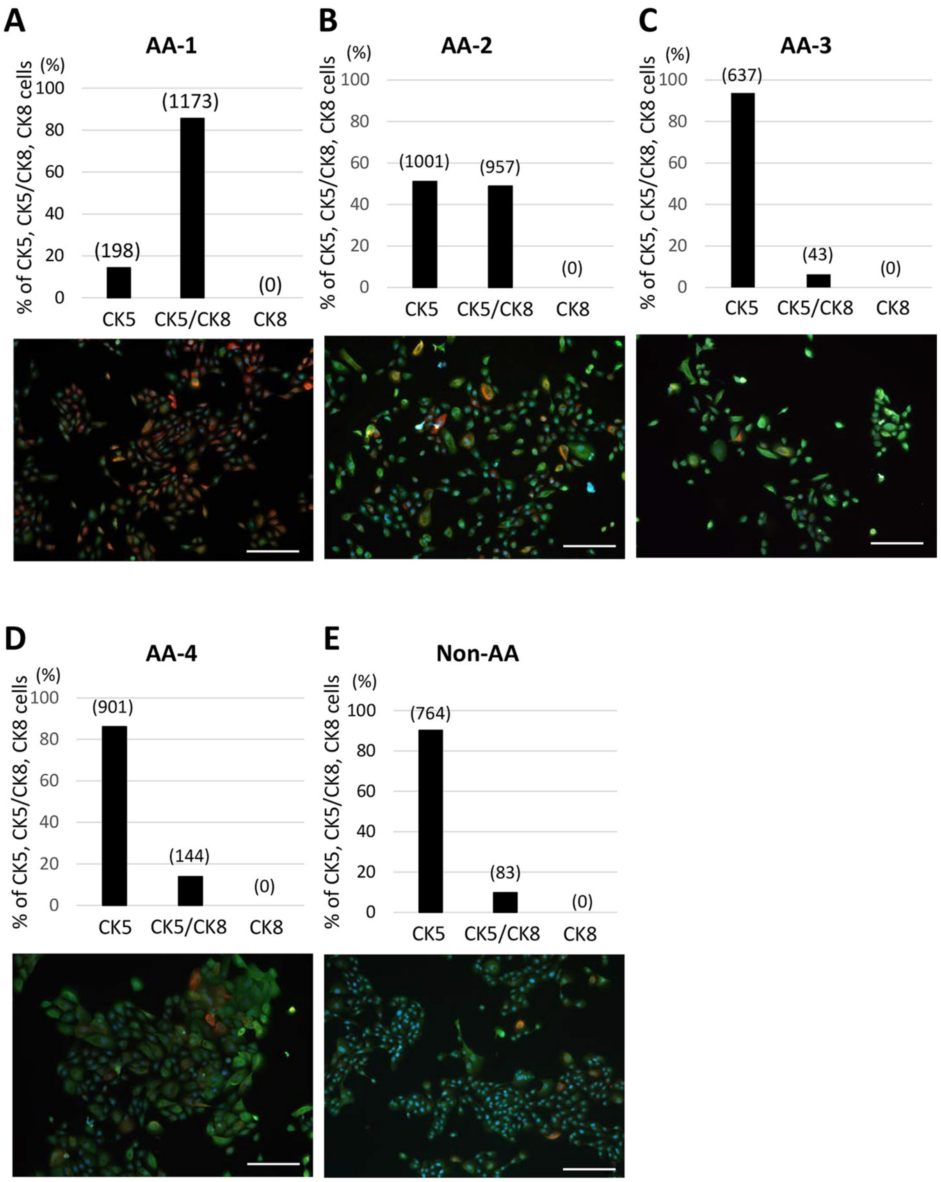

Supplementary Figure 2: Initial cell populations used for organoid culture in terms of luminal and basal cell marker expression. Prior to organoid culture, AA-1 cells **(A)**, AA-2 cells **(B)**, AA-3 cells **(C)**, AA-4 cells **(D)**, and Non-AA cells **(E)** were stained with anti-cytokeratin 5 (CK5) pAb (green) and anti-cytokeratin 8 (CK8) mAb (red). Double-staining for CK5 and CK8 shows basal (CK5; green), intermediate (CK5/CK8; yellow), and luminal (CK8; red) cell populations. The number in parentheses is the total cell number of each population. Representative images are shown. Scale bars 200 um.

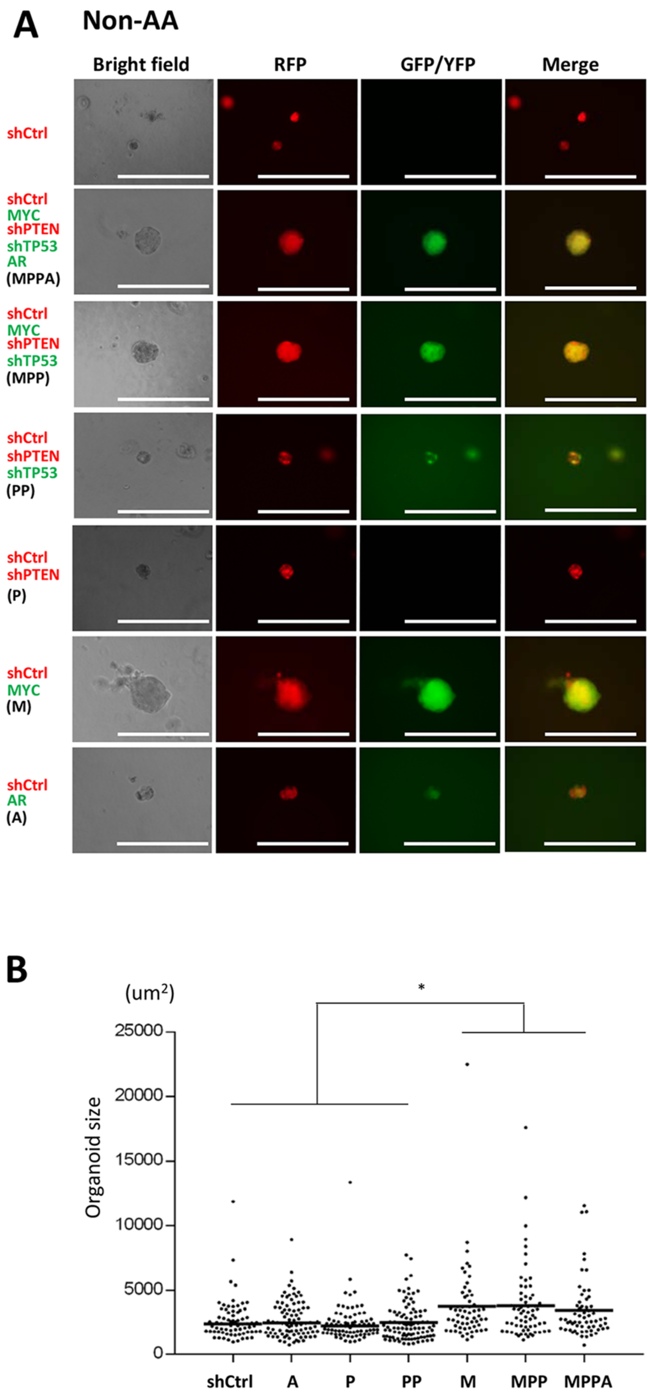

Supplementary Figure 3: The sizes of MPPA, MPP, PP, P, M, A, and shCtrl organoids derived from Non-African American tissue. **(A)** Representative bright field and fluorescence images of MPPA, MPP, PP, P, M, A, and shCtrl organoids at day 8. Scale bars 300 um. **(B)** Organoid sizes (areas) at day 8. The areas (um^2) of organoids >25 um diameter were measured by ImageJ. *$p < 0.05$.

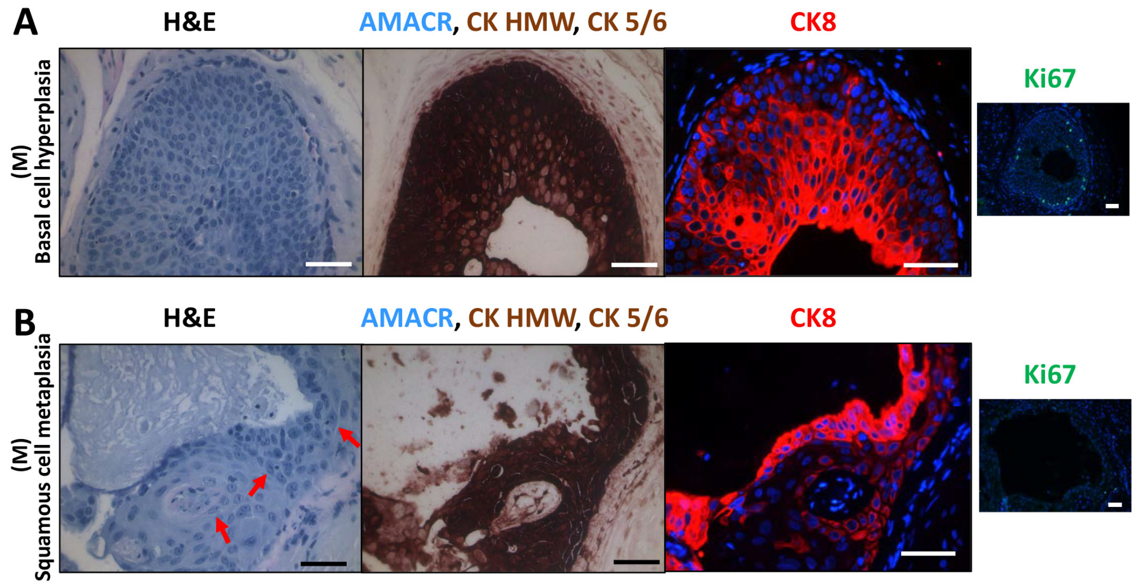

Supplementary Figure 4: *In vivo* development of transformed MYC-transduced (M) organoids. (A) Basal cell hyperplasia developed from M organoid transplants. Images with H&E staining; IHC staining for AMACR, CK HMW, and CK 5/6; and immunofluorescence staining for CK8 and Ki67. Blue, DAPI. **(B)** Squamous cell metaplasia developed from M organoid transplants. Images with H&E staining (Red arrows, squamous cells); IHC staining for AMACR, CK HMW, and CK 5/6; and immunofluorescence staining for CK8 and Ki67. Blue, DAPI. Scale bars 50 um.
